# Supplementary material for: A conserved enhancer regulates Il9 expression in multiple lineages
Source: Nat Commun. 2018 Nov 15;9:4803. doi: 10.1038/s41467-018-07202-0 (PMC6237898; doi:10.1038/s41467-018-07202-0)
Supplement: Supplementary file 1 — Supplementary Information [file 41467_2018_7202_MOESM1_ESM.pdf]

A conserved enhancer regulates *Il9* expression in multiple lineages

Koh et al

Supplementary Information

Supplementary Fig.1

a

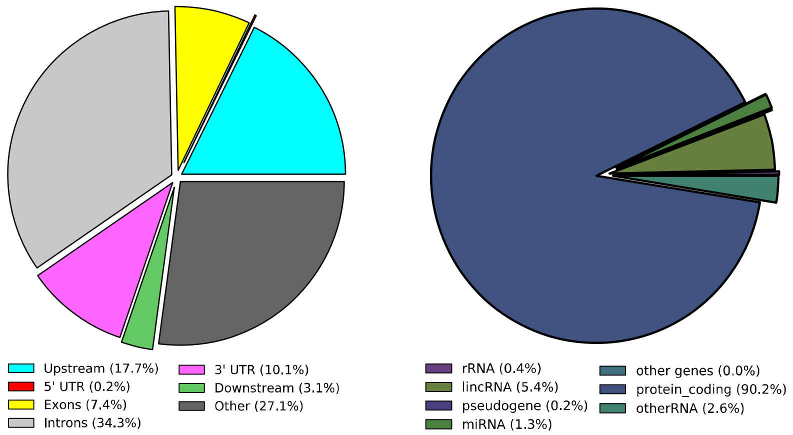

b

AGGACTGAAGGAAGAGAAATCCAGCAAGTTTCCAGTAAACAGTTTTTCCTTTGTTTAGCAGACCTTATAATTTGCTAACGAGTATCTTACAAGACTTC

AAAGGATGTCATGAGGCTTGTCTGCCAAGCAGCTGTTTTTTCTTAAACTGAACAAAAGAAACTGCAAGAGAAACCTCAGACCACAGGGGCTTGAA

GATTAGGAGTGAATGAGGTAACAGAAGATAGTGACTCTTGATACCAGTCCTCATCATGC*C*AATTCCATGAACTGCTTATGACAGGTCTATGGCGGTG

TAGACAGCAGATTTTCAGAGGTTCCATGTTGGGGCAAATCATGTGAAAGTGTGGGCACTCGGTGGGTTTAGTGACACAGTGAATAGAGTTTTTACAAAC

TCGAATGTTTGAGAAGGAGGAGGAGGAGGTGGGAAGGTTGCTCTGACAATCATTTAGTATGAGCTGTATCAGTCTCTCTGATCAGATTTTTTTCCTTTCA

AACCACAAACAGGATGCAGAAGACTTGTGCTGAGACAAAAAAAAATGTTTGGGTGCCA***GAAACAGATCA***CTAAAAGAGTGTGTTTTGCATAGCTTT

GTCAAAGCAGGCGACCACTTTAAAACTTGCCATTTTCTAGTTTTAAATTCAGTCATGACAAGCAATACGTCCAGTGATTCTTTACAGATATAAACACA

TGCTGAGAGTTGGCTTGCCCATCCTTTTATATGCAGGCATT

AICE
STAT
SMAD
IRF
GATA
E-box
ETS

Supplementary Fig.1

(a) Distribution of genome-wide p300 binding sites in Th9 cells relative to annotated features of known genes. Binding sites relative to gene structural segments (left) and classes of coding and non-coding RNAs (right) are indicated. Numbers indicate percentages. UTR, untranslated region. (b) Sequence of CNS-25. Putative binding sites for IL-9 inducing TFs binding sites are indicated.

Supplementary Fig.2

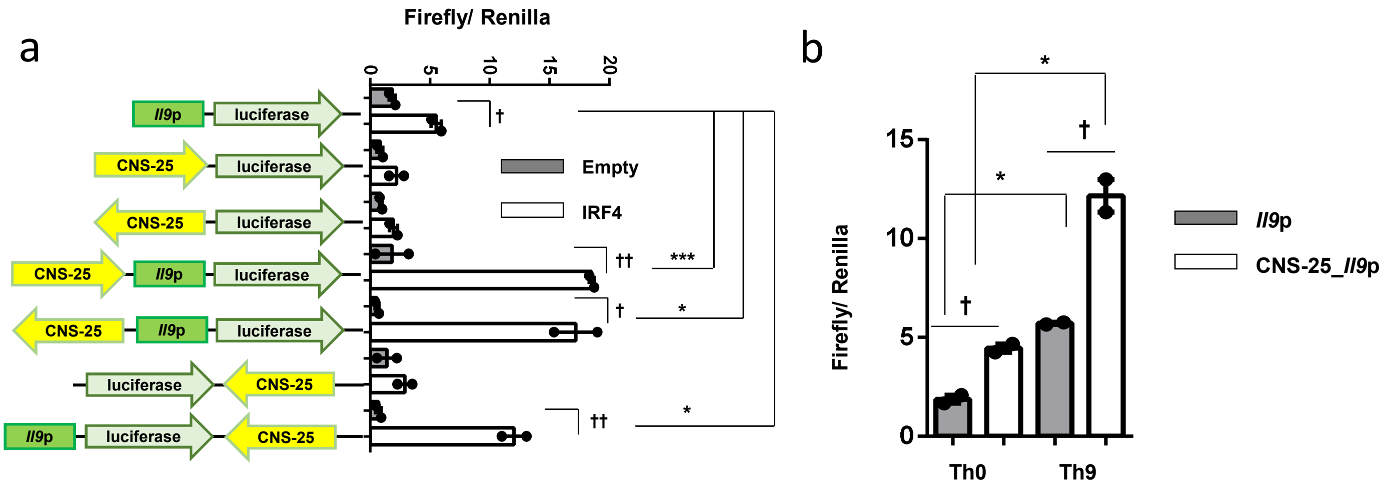

Supplementary Fig.2

EL4 T cells (a) or primary cultured T cells (b) were transfected with the *Il9* locus reporter vectors with or without IRF4-expressing vector. Reporter activities were calculated by a ratio of firefly luciferase over Renilla luciferase controls. (a, b) Data are represented as mean ± SEM from two independent experiments (n=2 per group). A two-tailed Student *t* test was used for pairwise comparisons. **p*< 0.05, ****p* <0.001, †*p*< 0.05, †† *p* <0.01.

Supplementary Fig.3

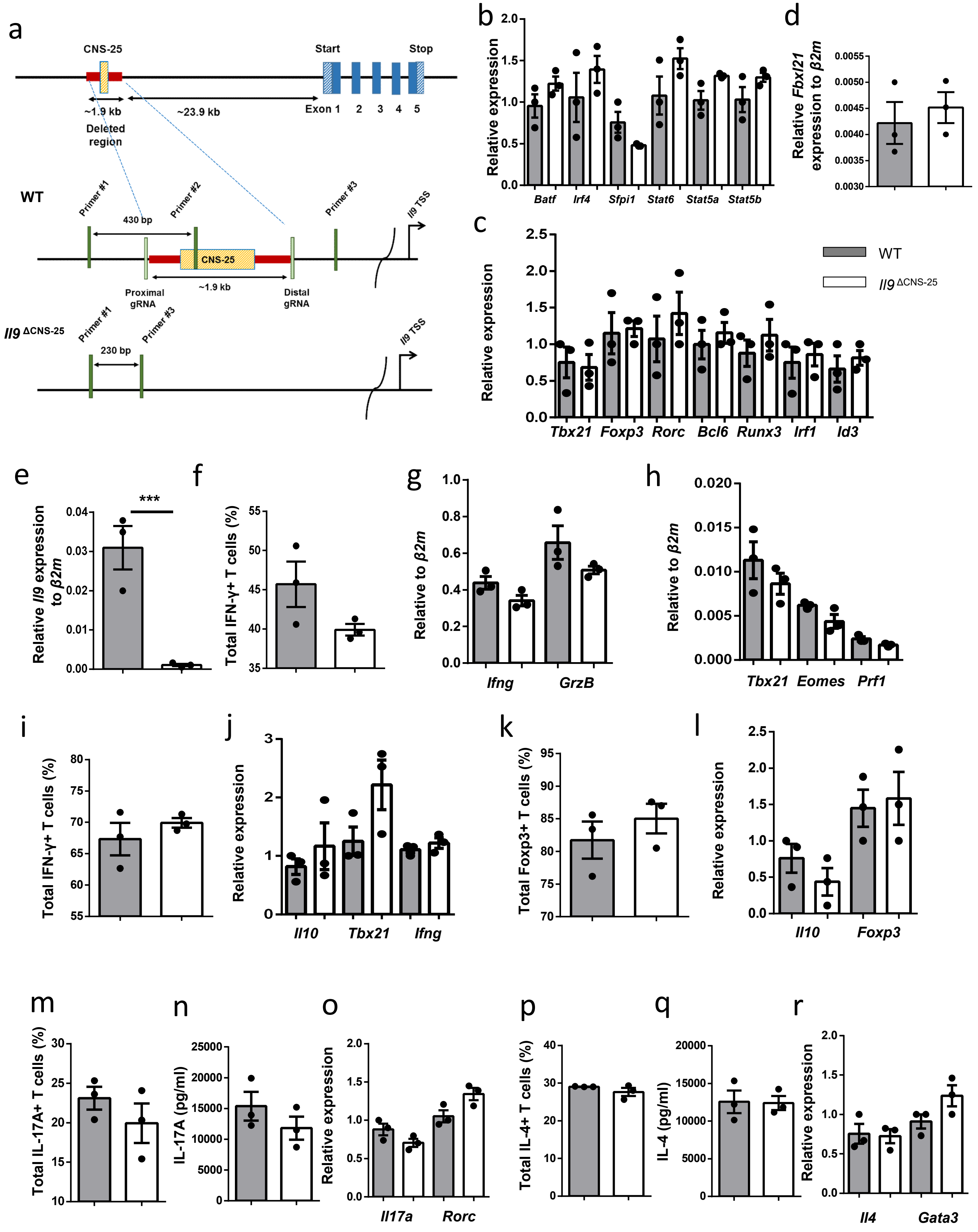

Supplementary Fig.3

(a) Strategy for generating *Il9* $^{\Delta CNS\text{-}25}$ mice. Mouse *Il9* genomic locus (top) and *Il9* CNS-25 targeting gRNAs and genotyping primer set for detecting WT allele: Primer #1 and #2 (middle), were shown. Mouse *Il9* locus after CRISPR/Cas9 mediated gene editing and genotyping primer set for detecting CNS-25 deletion allele: Primer #1 and #3 (bottom). (b, c) The expression of IL-9-inducing TFs (b) or -repressing TFs (c) in WT or *Il9* $^{\Delta CNS\text{-}25}$ Th9 cells. (d) *Fbxl21* expression or brain in WT or *Il9* $^{\Delta CNS\text{-}25}$ mice. (e-h) The expression of *Il9* and (e) IFN-γ production (f) of Tc9 cells was assessed by qRT-PCR and intracellular staining, respectively. The expression of cytokines, TFs and enzymes (g, h) was assessed by qRT-PCR. (i, j) The percentage of IFN-γ producing T cells (i) and the expression of *Il10*, *Tbx21* and *Ifng* (j) in WT or *Il9* $^{\Delta CNS\text{-}25}$ Th1 cell cultures. (k, l) The percentage of Foxp3 positive T cells (k) and the expression of *Il10* and *Foxp3* (l) in WT or *Il9* $^{\Delta CNS\text{-}25}$ Treg cell cultures. (m-o) The percentage of IL-17A producing T cells (m), the amount of IL-17A (n) and the expression of *Il17a* and *Rorc* (o) in WT or *Il9* $^{\Delta CNS\text{-}25}$ Th17 cell cultures. (p-r) The percentage of IL-4 producing T cells (p), the amount of IL-4 (q) and the expression of *Il4* and *Gata3* (r) in WT or *Il9* $^{\Delta CNS\text{-}25}$ Th2 cell cultures. All gene expression was measured using qRT-PCR and normalized to *β2m* expression. Data are represented as mean ± SEM from three independent experiments (n=3 per group). A two-tailed Student *t* test was used for pairwise comparisons. **p*< 0.05, ***p*<0.01, ****p* <0.001.

Supplementary Fig.4

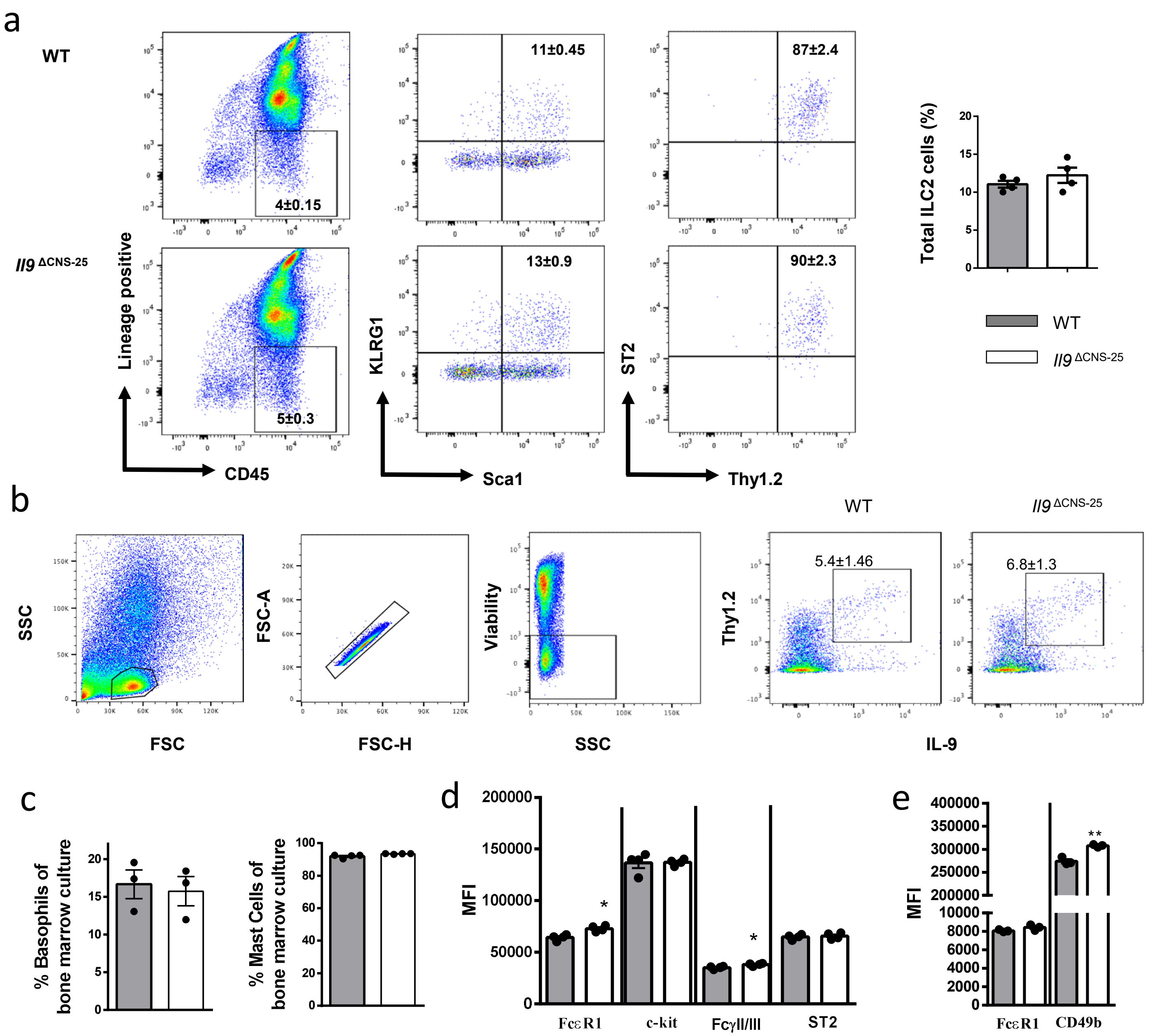

Supplementary Fig.4

(a) Scheme for analysis of ILC2s. Total lung cells were stained with lineage positive staining cocktail and other ILC2 specific markers. (b) Scheme for analysis of ILCs. Total lung cells were lineage-depleted before analysis as indicated in the panel. (c) Frequencies of mast cells (21 days) and basophils (7 days) differentiated in culture using IL-3 and SCF for mast cells or IL-3 alone for basophils. (d) MFI of surface receptors on mast cells differentiated in IL-3 and SCF for 21 days. (e) MFI of surface receptors for basophils differentiated in IL-3 for 7 days as determined by flow cytometry. All gene expression was measured using qRT-PCR and normalized to *β2m* expression. Data are represented as mean ± SEM from three independent experiments (n=3-4 per group). A two-tailed Student *t* test was used for pairwise comparisons. **p*< 0.05, ***p*<0.01, ****p* <0.001.

Supplementary Fig.5

a

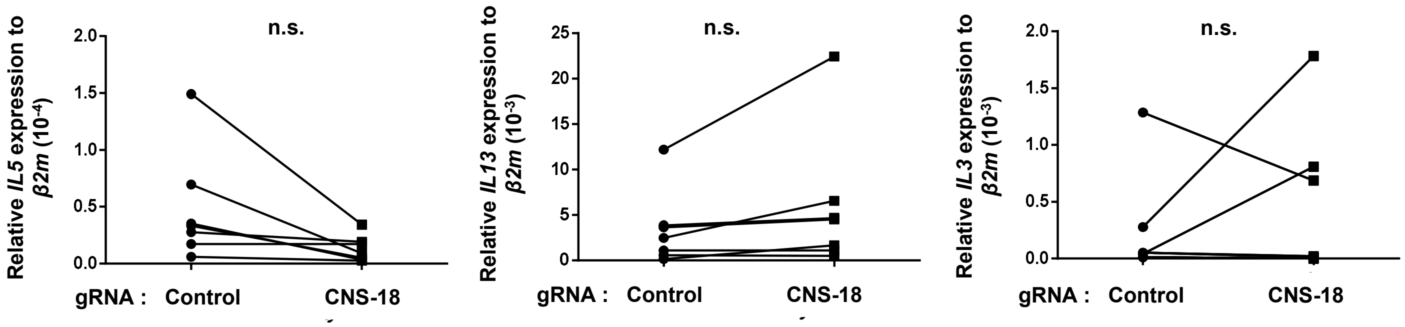

b

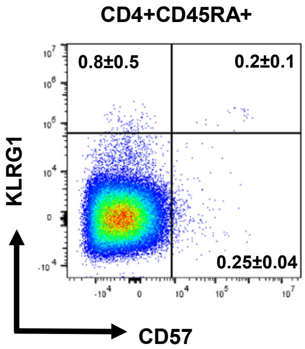

Supplementary Fig.5

(a) The qRT-PCR analysis of *IL5, IL13* and *IL3* in Th9 cells transduced with control or CNS-18-targeting lentivirus. The gene expression and deletion efficiency of *IL9* CNS-18 were normalized to *β2m* expression and amplification in -12kb sequence respectively. Data are mean ± SEM of 7 donors. n.s., not significant.

(b) Analysis of the purity of human naïve T cells after magnetic separation. Data are mean ± SEM of 3 donors.

Supplementary Table 1. Sequences of mouse *Il9* CNS-25 targeting gRNAs and *Il9* $^{\Delta CNS\text{-}25}$ mice genotyping primers

<table>
<tr><th>CNS-25 mice</th><th colspan="3">Protospacer sequence (5’-3’)</th></tr>
<tr><td>Il9 CNS-25 KO 5’
(Proximal gRNA)</td><td colspan="3">CAATCACCTAGCTAACTCGG</td></tr>
<tr><td>Il9 CNS-25 KO 3’
(Distal gRNA)</td><td colspan="3">TGCATTCGAGTCCCCAAATG</td></tr>
<tr><th>Genotyping primers</th><th>primer sequence (5’-3’)</th><th colspan="2">Program</th></tr>
<tr><td rowspan="2">CNS-25 primer #1</td><td rowspan="2">AAACCATTGTGTGATGTACCTGG</td><td colspan="2">95°C 5 mins</td></tr>
<tr><td>95°C 30 sec</td><td rowspan="3">X 34
cycle</td></tr>
<tr><td rowspan="2">CNS-25 primer #2</td><td rowspan="2">AAGCCTCATGACATCCTTTGA</td><td>58 °C 30 sec</td></tr>
<tr><td>72 °C
1 min</td></tr>
<tr><td rowspan="2">CNS-25 primer #3</td><td rowspan="2">AACTGACCAGATTTACTAGGTCCC</td><td colspan="2">72 °C 10 mins</td></tr>
<tr><td colspan="2">4 °C overnight</td></tr>
</table>

Supplementary Table 2. CRISPR/Cas9 plasmids

Construct	Oligo	Overhang	Protospacer	Overhang
New PX330A_D10A 1X2	sgRNA-1 sense	5’-CACCG	(N)20	
	sgRNA-1 antisense	3’-C	(N)20 complement	CAAA-5’
PX330S_2	sgRNA-1 sense	5’-CACCG	(N)20	
	sgRNA-1 antisense	3’-C	(N)20 complement	CAAA-5’

Supplementary Table 3. Sequences of gRNAs targeting *hIL9* CNS-18 and GM38602

gRNAs	Target location	Protospacer sequence (5’-3’)	Genome
hIL9 CNS-18 5A	CNS-18	CAGAGTAGATCTTCCATTGG	Human
hIL9 CNS-18 3A	CNS-18	AGAGATGGGGTCTCCCTATG	Human
mGM38602 5A	GM38602	GCCCGGGCTCGATCTATTAA	Mice
mGM38602 3A	GM38602	CCTCTCCAGGTGTTAACTAC	Mice

Supplementary Table 4. Taqman probes for qPCR

Mouse	
Gene	Cat. No.
β2m	Mm00437762_m1
β-actin	Mm02619580_g1
Batf	Mm00479410_m1
Bcl6	Mm00477633_m1
Eomes	Mm01351985_m1
Foxp3	Mm00475165_m1
Gata3	Mm00484683_m1
GrzB	Mm00442834_m1
Id3	Mm00492575_m1
Ifng	Mm01168134_m1
Il10	Mm00439614_m1
Il17a	Mm00439618_m1
Il4	Mm00445259_m1
Il6	Mm99999604_m1
Il9	Mm00434305_m1
Irf1	Mm00515191_m1
Irf4	Mm00516431_m1
Junb	Mm04243546_s1
Prf1	Mm00812512_m1
Rorc	Mm00441139_m1
Runx3	Mm00490666_m1
Sfpi1	Mm00488142_m1
Stat6	Mm01160477_m1
Tbx21	Mm00450960_m1
Human	
Gene	Cat. No.
B2M	Hs99999907_m1
IL10	Hs99999035_m1
IL21	Hs00222327_m1
IL9	Hs00914237_m1

Supplementary Table 5. Sequences of eRNA primers and HBB-bs

Human		
Primers	Forward (5'-3')	Reverse (5'-3')
Hbb-bs	GAGTGGCACAGCATCCAGGGAGAAA	CCACAGGCCAGAGACAGCAGCCTTC
e1	AAGTGGCCCCAACTTACAGA	CGCTTGCAGACACCTTCAAA
e2	TGCCACCAAAGCTAATGACC	ACACACACACACACACCATG
e3	TCTAGAGAGCACCCCAAACT	GTTTACTGAGGCTTGTTGATTGA
e4	CATTGTGTGATGTACCTGGGG	CCCTGACAAGTTCACTGTGC
e5	GTTGACTGGAACCACTGCTG	CAGACCAAGCAAGCTACCCT
e6	CTGCTTGGACTGTTTCACCC	CTTCCTCTTCCCTCCCTTCC

Supplementary Table 6. Fluorescent antibodies for flow cytometric analysis

<table>
<tr><th>Antigen/Name</th><th>Clone</th><th>Fluorochrome</th><th>Company</th><th>Cat. No.</th></tr>
<tr><td>B220</td><td>HIS24</td><td>FITC</td><td>BD Biosciences</td><td>561876</td></tr>
<tr><td rowspan="2">CD11b</td><td rowspan="2">M1/70</td><td>PerCP-Cy5.5</td><td>eBioscience</td><td>45-0112-82</td></tr>
<tr><td>FITC</td><td>BD Biosciences</td><td>557396</td></tr>
<tr><td>CD11c</td><td>N418</td><td>PE-Cy7</td><td>eBioscience</td><td>25-0114-82</td></tr>
<tr><td>CD16/32</td><td>19</td><td>PerCP-Cy5.5</td><td>BioLegend</td><td>101323</td></tr>
<tr><td rowspan="2">CD3</td><td rowspan="2">145-2C11</td><td>PerCP-Cy5.5</td><td rowspan="2">BD Biosciences</td><td>551163</td></tr>
<tr><td>FITC</td><td>553061</td></tr>
<tr><td rowspan="6">CD4</td><td rowspan="6">GK1.5</td><td>FITC</td><td>BD Biosciences</td><td>553729</td></tr>
<tr><td>PE</td><td>BioLegend</td><td>100408</td></tr>
<tr><td>PerCP-Cy5.5</td><td>BioLegend</td><td>100434</td></tr>
<tr><td>PE-Cy7</td><td>BioLegend</td><td>100422</td></tr>
<tr><td>APC</td><td>BioLegend</td><td>100412</td></tr>
<tr><td>APC-Cy7</td><td>BD Biosciences</td><td>552051</td></tr>
<tr><td>CD49b</td><td>HMα2</td><td>PE</td><td>BioLegend</td><td>103506</td></tr>
<tr><td rowspan="3">c-Kit</td><td rowspan="3">2B8</td><td>FITC</td><td rowspan="3">BioLegend</td><td>105805</td></tr>
<tr><td>APC</td><td>105811</td></tr>
<tr><td>APC-Cy7</td><td>105825</td></tr>
<tr><td>F4/80</td><td>BM8</td><td>FITC</td><td>BioLegend</td><td>123108</td></tr>
<tr><td rowspan="4">FcεR1</td><td rowspan="4">MAR-1</td><td>FITC</td><td rowspan="4">BioLegend</td><td>134305</td></tr>
<tr><td>PE</td><td>134307</td></tr>
<tr><td>PE-Cy7</td><td>134317</td></tr>
<tr><td>APC</td><td>134315</td></tr>
<tr><td>Foxp3</td><td>MF23</td><td>FITC</td><td>BD Biosciences</td><td>560403</td></tr>
<tr><td>IFN-γ</td><td>XMG 1.2</td><td>PerCP-Cy5.5</td><td>eBioscience</td><td>45-7311-82</td></tr>
<tr><td>IL-17A</td><td>eBio17B7</td><td>PE-Cy7</td><td>eBioscience</td><td>25-7177-82</td></tr>
<tr><td>IL-4</td><td>11B11</td><td>AF647</td><td>BioLegend</td><td>504110</td></tr>
<tr><td>IL-9</td><td>RM9A4</td><td>PE</td><td>BioLegend</td><td>514104</td></tr>
<tr><td>LAMP-1</td><td>DATK32</td><td>APC</td><td>BD Biosciences</td><td>562376</td></tr>
<tr><td rowspan="2">Ly6G</td><td>1A8</td><td>APC</td><td>BioLegend</td><td>127613</td></tr>
<tr><td>RB6-8C5</td><td>FITC</td><td>BD Biosciences</td><td>553126</td></tr>
<tr><td>NK 1.1</td><td>PK136</td><td>FITC</td><td>BD Biosciences</td><td>553164</td></tr>
<tr><td>SiglecF</td><td>E50-2440</td><td>PE</td><td>BD Biosciences</td><td>552126</td></tr>
<tr><td>ST2</td><td>U29-93</td><td>PE</td><td>BD Biosciences</td><td>566309</td></tr>
<tr><td>Ter119</td><td></td><td>FITC</td><td>BioLegend</td><td>116205</td></tr>
<tr><td>Fixable Viability dye</td><td></td><td>eFluor 780</td><td>eBioscience</td><td>65-0865-14</td></tr>
</table>

Supplementary Table 7. ELISA capture and biotinylated secondary antibodies

Capture Abs	Clone	Stock	Final conc.	Company	Cat. No.
IL-17A	TC11-18H10	0.5mg/ml	2ug/ml	BD Biosciences	555068
IL-10	JES5-2A5	1mg/ml	4ug/ml	BD Biosciences	551215
IL-4	11B11	0.5mg/ml	2ug/ml	BD Biosciences	554434
IFN-γ	R4-6A2	1mg/ml	2ug/ml	BD Biosciences	551216
Secondary Abs	Clone	Stock	Final conc.	Company	Cat. No.
IL-17A	TC11-18H10	0.5mg/ml	1ug/ml	BD Biosciences	555067
IL-10	SXC-1	0.5mg/ml	1ug/ml	BD Biosciences	554423
IL-4	BVD6-24G2	0.5mg/ml	1ug/ml	BD Biosciences	554390
IFN-γ	XMG1.2	0.5mg/ml	1ug/ml	BD Biosciences	554410
Target	Description			Company	Cat. No.
IL-6	Mouse IL-6 ELISA MAX Deluxe			BioLegend	431304
IL-9	Mouse IL-9 ELISA MAX Deluxe			BioLegend	442704

Supplementary Table 8. Antibodies for ChIP assay

Transcription factor				
Antigen/Name	Clone	Host	Company	Cat. No.
BATF	D7C5	Rabbit	Cell Signaling Technology	8638
CRSP1/TRAP220	polyclonal	Rabbit	Bethyl Laboratories	A300-793A
CTCF	D31H2	Rabbit	Cell Signaling Technology	3418
Foxo1	polyclonal	Rabbit	Abcam	ab39670
GATA3	D13C9	Rabbit	Cell Signaling Technology	Sc-268
IRF4	D9P5H	Rabbit	Cell Signaling Technology	15106
	H-140	Rabbit	Santa Cruz Biotechnology	Sc-28696
p300	N-15	Rabbit	Santa Cruz Biotechnology	Sc-584
PU.1	9G7	Rabbit	Cell Signaling Technology	2258
SMC1	polyclonal	Rabbit	Bethyl Laboratories	A300-055A
STAT5	C-17	Rabbit	Santa Cruz Biotechnology	Sc-835
STAT6	M-20	Rabbit	Santa Cruz Biotechnology	Sc-981
Histone modification				
Antigen/Name	Clone	Host	Company	Cat. No.
H3K27ac	polyclonal	Rabbit	Abcam	ab4729
H3K27me3	mAbcam6002	mouse	Abcam	ab6002
H3K4me1	polyclonal	Rabbit	Abcam	ab8895
H3K4me3	polyclonal	Rabbit	Abcam	ab8580
Normal Rabbit IgG	polyclonal	Rabbit	Millipore	12-370

Supplementary Table 9. Sequences of ChIP primers

Mouse		
Primers	Forward (5'-3')	Reverse (5'-3')
Il9 CNS-6<br>-6 kb	GAGCTGAACGCAGGCCAAGAACGA	CTTGGAACTAGTTATCTCTCCACTG
Il9 promoter<br>-5bp ~ -67bp	GTGGGCACTGGGTATCAGTTTGATGT	CAGTCTACCAGCATCTTCCAGTCTAG
Il9 CNS -25_1	ATGTCATGAGGCTTGTCTGC	ACTCCTAATCTTCAAGCCCCT
Il9 CNS -25_2	AGCAGGCGACCACTTTAAAA	GCCAACTCTCAGCATGTGTT
Il9 -35 kb	GAGGGAGAGGGGAAAACACA	TACCGCTCCGCAGTCTAAAT
Il9 -12 kb	GTTGCCTTGGTTATGGTGCT	AGAATGGCCCATGAAGACCA
Human		
Primers	Forward (5'-3')	Reverse (5'-3')
hIL9 CNS-4.5	GTCACCTCACCTGTCTCCTT	ACATTGGTGCAGGGTTTGAG
hIL9 promoter	AAGTGGCCCCAACTTACAGA	CGCTTGCAGACACCTTCAAA
hIL9 CNS -18_1	ACCTAGCCCACTGTGCAACT	CATGATGACCCTGTGGTCTG
hIL9 CNS -18_2	TTTCAGAGTCAGAAGAAAAGATGG	CATTTAGGGTGTTGCCTTTCA
hIL9 -30 kb	AGACCAAGGACGTTAGAGCA	GTTGCCATTTTAGCTAGCTTTGG
hIL9 -12 kb	CTGGGCTCTTTGGAGAAATG	CAATGTGGCTTTTGGGATTT
